# Linking signal integrity to probabilistic models of behavioral dynamics

**DOI:** 10.3758/s13428-026-03132-8

**Published:** 2026-08-18

**Authors:** Reza Sayfoori, Hung Cao

**Affiliations:** 1 Department of Electrical Engineering and Computer Science, University of California, Irvine, Irvine, CA 92697 USA; 2 Department of Biomedical Engineering, University of California, Irvine, Irvine, CA 92697 USA; 3 Department of Computer Science, University of California, Irvine, Irvine, CA 92697 USA

**Keywords:** Ultra-wideband (UWB), Behavioral phenotyping, Markov chains, Information-theoretic analysis, Signal integrity, Quantitative ethology, Sensor-based behavioral modeling

## Abstract

Quantitative analysis of rodent behavior in naturalistic settings is crucial for neuroscience, yet traditional methods often lack precision or scalability. While ultra-wideband (UWB) sensor tracking provides centimeter-level localization, standard metrics like root mean square error (RMSE) fail to capture the probabilistic and sequential nature of behavior. We introduce a probabilistic, information-theoretic framework that leverages high-resolution UWB sensor trajectories to address this gap. By integrating Shannon entropy to quantify uncertainty, Bernoulli modeling to assess accuracy thresholds, and first-order Markov chains to characterize state dynamics, our approach derives interpretable behavioral markers directly linked to signal quality. Empirical evaluation in an open-field arena demonstrated robust tracking under line-of-sight (LoS; RMSE: 20 mm) and non-line-of-sight (NLoS; RMSE: 35 mm) conditions. Crucially, we show that physical-layer impairments propagate to behavioral metrics: NLoS conditions increased entropy from 1.15 to 1.78 bits, reduced the probability of achieving sub-20 mm accuracy from 63.2% to 27.7%, and decreased state persistence, indicating greater behavioral fragmentation. By treating UWB signals as a probabilistic information source, our computationally efficient framework establishes a methodological bridge between engineering performance and neuroscience, enabling scalable, reproducible, and low-bias behavioral quantification suitable for preclinical research in complex environments.

## Introduction

Understanding behavior within complex, naturalistic environments is a fundamental goal in neuroscience, providing critical insights into neural function and dysfunction, particularly how experience shapes neural circuits (Polley, Kvašňák, & Frostig, 2004). Rodents, as key experimental models for neurological disorders and therapeutic evaluation, offer a valuable platform for these investigations (Durbin, Eddy, Krogh, & Mitchison, 1998). However, accurately quantifying their behavior in settings that mimic natural habitats presents significant challenges (Rabiner, 1989). While automated tracking systems have advanced beyond labor-intensive manual scoring, many current approaches focus narrowly on simple metrics like spatial occupancy or gross locomotion (Jurafsky & Martin, 2019). These methods often neglect the rich temporal dependencies, probabilistic structure (Papoulis & Pillai, 2002), and context-driven transitions that shape complex behavioral sequences, particularly in more naturalistic scenarios (Patterson, Thomas, Wilcox, Ovaskainen, & Matthiopoulos, 2008). Such limitations hinder the development of sensitive behavioral assays needed for translational research.

These shortcomings reduce sensitivity to subtle phenotypic differences, hinder early detection of behavioral biomarkers, and obscure inter-individual variability (Anderson & Johnson, 2015). In translational neuroscience, where reproducibility and sensitivity are essential, such limitations are critical. To address them, we extend ultra-wideband (UWB) sensor tracking beyond its typical application for localization accuracy. UWB sensor delivers centimeter-level spatial precision, high temporal resolution, and robustness against multipath interference, minimally constraining naturalistic behavior (Guo, Chen, & Li, 2021; Molisch, 2011). Yet, most UWB signal and sensor studies emphasize deterministic accuracy metrics (e.g., RMSE, SNR), failing to exploit the richer probabilistic and temporal information embedded within the high-resolution trajectories (Sayfoori et al., 2025). This gap limits the translation of precise positional data into systematic models of behavioral variability and dynamics.

We introduce a probabilistic and information-theoretic framework that leverages UWB sensor tracking to overcome these limitations. Shannon entropy (Shannon, 1948) quantifies uncertainty in error distributions and behavioral transitions (Schreiber, 2000), Bernoulli modeling estimates the probability of meeting accuracy thresholds, and first-order Markov chains characterize sequential state dynamics (Bush & Larkins, 2005; Wiltschko et al., 2015). First formalized by Andrey Markov (Markov, 1906), these computationally efficient models uncover latent temporal structure and quantify variability, providing probabilistic state representations (Bush & Larkins, 2005). While machine learning approaches exist (Luxem et al., 2022; Pereira, Shaevitz, & Murthy, 2020), they often require extensive annotated datasets and lack interpretability, whereas Markov models offer a balance of simplicity and transparency well-suited for moderate datasets (Bush & Larkins, 2005). By integrating these complementary analyses, our framework transforms continuous UWB sensor trajectories into interpretable behavioral markers that link system-level accuracy with biological meaning. For example, elevated entropy in exploratory transitions may indicate fragmented search strategies in anxiety models, while persistence probabilities can reflect drug-induced stabilization of feeding–resting cycles.

To address the gap in current UWB sensor applications, we propose an integrated framework combining UWB sensor RTLS with these information-theoretic and sequential analyses. Continuous trajectories are discretized into ethologically relevant states—resting, exploring, feeding, and burrowing—and transition matrices are constructed to quantify behavioral dynamics. To the best of our knowledge, this is the first application of an entropy-, Bernoulli-, and Markov-based probabilistic toolkit specifically within a UWB tracking system, establishing a physics-to-behavior pipeline that unifies communication theory with behavioral neuroscience.

This study builds directly upon our earlier work published recently (Sayfoori et al., 2025), where we validated the technical accuracy of UWB localization under LoS and NLoS conditions using deterministic metrics like RMSE and SNR. While that work confirmed system reliability, it did not address how signal-level impairments affect behavioral interpretation. The present study extends that foundation by applying the proposed probabilistic framework—integrating Shannon entropy, Bernoulli success modeling, and Markov chain analysis—to the UWB-derived trajectories. In doing so, we reframe UWB not merely as a localization technology but as a probabilistic information source capable of nuanced behavioral phenotyping, thereby complementing and significantly extending our prior findings.

## Methods

This study develops and validates a probabilistic framework for modeling rodent behavioral dynamics using high-precision UWB sensor localization data. Building upon our earlier work that focused on deterministic localization accuracy metrics (Sayfoori et al., 2025), the present analysis reuses a previously validated dataset and shifts the emphasis from localization fidelity alone to probabilistic behavioral interpretation. The analysis proceeded in the following order: first, UWB localization performance was characterized under LoS and NLoS signal conditions; second, continuous (*x*, *y*) trajectories were converted into discrete behavioral states using predefined spatial and kinematic criteria; third, localization reliability was quantified using error entropy and Bernoulli threshold modeling; fourth, behavioral-state sequences were modeled using first-order Markov chains; and finally, model reproducibility was evaluated using prediction accuracy, stationary-distribution agreement, entropy rate, persistence probabilities, and Frobenius-distance comparisons.

### Experimental setup

Experiments were conducted in a custom-built open-field arena (122×61 cm), constructed from epoxy-coated glass to minimize multipath reflections and electromagnetic interference (Sayfoori et al., 2025). Eight UWB sensor anchors (Decawave sensors, MDEK1001 sensor development boards with DWM1001-DEV sensor modules) were distributed around the perimeter following geometric dilution of precision (GDOP) analysis, ensuring optimal LoS coverage and minimizing positional bias. Anchor coordinates were measured and calibrated through GDOP-based optimization, yielding reproducible spatial geometry across sessions.

**Fig. 1:**
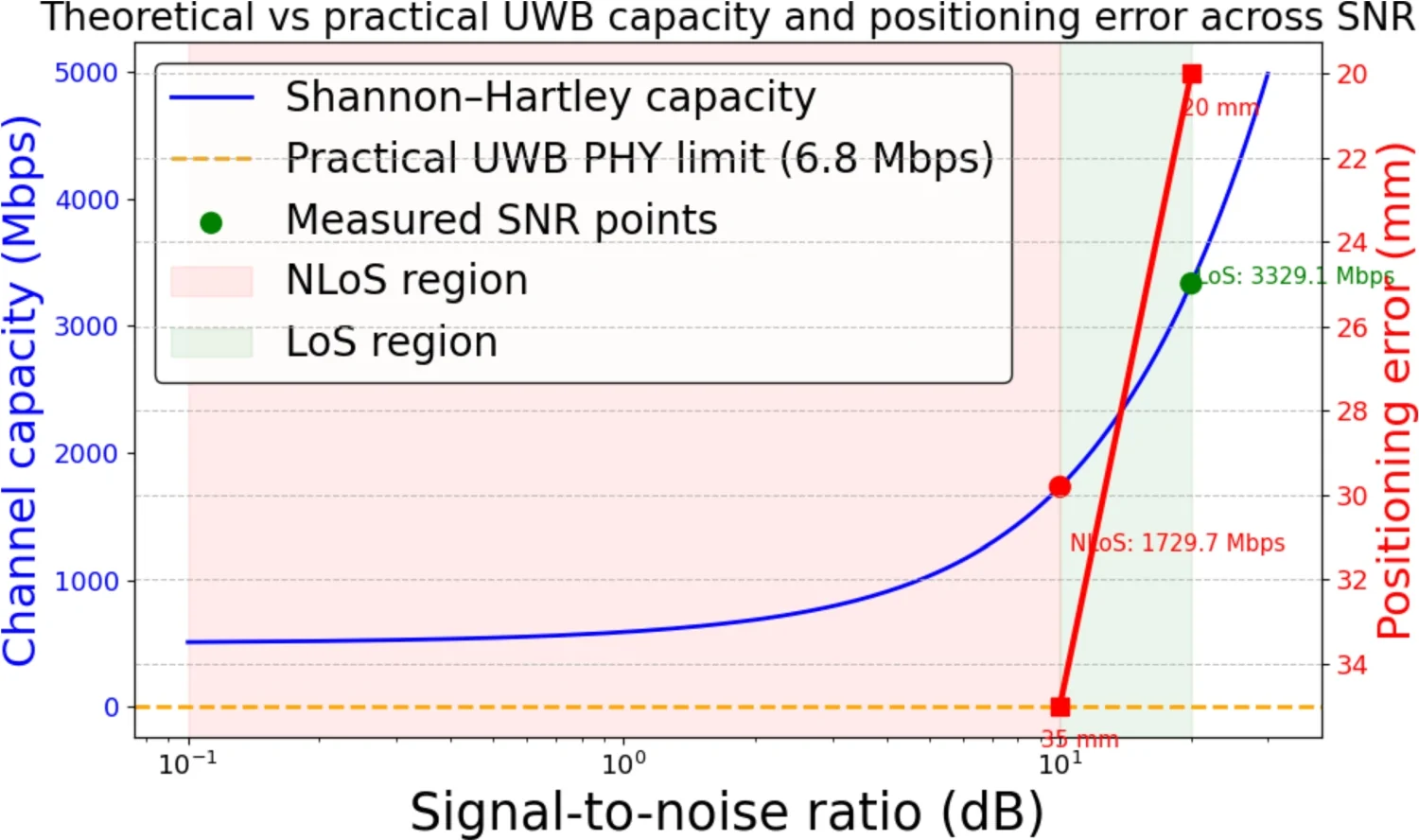
Theoretical Shannon–Hartley channel capacity compared with measured UWB sensor positioning errors under LoS and NLoS conditions. LoS preserves high capacity with low error, whereas NLoS reduces capacity and increases error, illustrating how channel quality propagates into localization accuracy

The UWB sensor system employed a symmetric two-way ranging (TWR) protocol. For each anchor–tag pair, distance *d* was estimated from the round-trip time-of-flight (ToF) as

1 $$\begin{aligned} d = \frac{c \; \Delta t}{2}, \end{aligned}$$

where *c* is the speed of light and Δt is the measured round-trip propagation delay. To mitigate clock offsets and measurement noise, an extended Kalman filter (EKF) (Kalman, 1960) was applied to sequential distance estimates. Filtered ranges were fused across anchors using trilateration to recover two-dimensional positions.

The rodent was equipped with a lightweight UWB sensor tag (≈20–25 grams including housing and battery) mounted on a custom-fabricated harness designed to minimize discomfort and behavioral interference. The animal was habituated to the harness and arena across multiple sessions before formal experiments to promote naturalistic activity. Localization accuracy was benchmarked against a synchronized ground-truth video tracking system. Under LoS conditions, mean positional errors were approximately 20 mm, while controlled NLoS conditions produced approximately 35 mm of error (Sayfoori et al., 2025). Position data were sampled at 10 Hz and logged for offline analysis. This dataset, initially collected for UWB sensor validation (Sayfoori et al., 2025), was reanalyzed here to extract behavioral structure using probabilistic and information-theoretic methods.

The reanalyzed dataset consisted of four 5-min tracking sessions obtained from one rodent and sampled at 10 Hz, yielding 3000 position samples per session. Two sessions were analyzed under LoS conditions and two sessions under controlled NLoS conditions. No pharmacological, disease-model, treatment, or multi-animal subject groups were introduced in the present reanalysis; therefore, “group” refers only to signal condition (LoS versus NLoS), not to independent animal cohorts. NLoS recordings corresponded to sessions in which direct signal propagation between the mobile tag and one or more anchors was partially obstructed by a physical barrier or arena obstruction, whereas LoS recordings preserved direct anchor–tag visibility. This design allowed the same probabilistic workflow to be evaluated under two signal-quality regimes while avoiding additional biological manipulations. Because the present work reanalyzes an existing localization dataset from a single-animal proof-of-concept study, session order was not treated as an experimental factor and formal counterbalancing was not performed; this limitation is acknowledged when interpreting condition-level comparisons.

### Signal integrity modeling

To contextualize localization fidelity, the Shannon–Hartley theorem (Shannon, 1948) was employed to estimate an upper bound on UWB sensor link capacity:

2 $$\begin{aligned} C = B \, \log _{2}\!\left( 1 + \frac{S}{N} \right) , \end{aligned}$$

where *C* is the channel capacity (bits/second), *B* is the effective bandwidth (Hz), and *S*/*N* is the signal-to-noise ratio (SNR).

In practical deployments Fig. 1, multipath propagation, antenna misalignment, and partial occlusions degrade the effective SNR, which in turn increases positioning error. This effect can be characterized through the variance of the estimation error, $\sigma _e^2$, which is inversely proportional to SNR:

3 $$\begin{aligned} \sigma _e^{2} \;\propto \; \frac{1}{\textrm{SNR}}, \end{aligned}$$

a relationship consistent with the Cramér–Rao lower bound (CRLB) for time-of-flight (ToF)–based ranging estimators. Here, $\sigma _e^2$ denotes the variance of the localization estimation error, and $\textrm{SNR}$ is the signal-to-noise ratio as defined below.

The instantaneous SNR was computed as

4 $$\begin{aligned} \textrm{SNR}(t) = \frac{P_{\text {signal}}(t)}{P_{\text {noise}}(t)}, \end{aligned}$$

where $P_{\text {signal}}(t)$ and $P_{\text {noise}}(t)$ denote signal and noise power at time *t*, respectively. Session-averaged SNR values were then compared across LoS and NLoS conditions, providing a physical basis for observed differences in entropy and trajectory variability.

### Behavioral state segmentation

Continuous UWB sensor trajectories were discretized into four ethologically meaningful states: *resting*, *exploring*, *feeding*, and *burrowing*. These states represent fundamental rodent activities relevant to preclinical neuroscience and ethology. Segmentation combined spatial zoning with kinematic thresholds to ensure ecological validity and compatibility with the 10-Hz sampling rate.

Velocity at time *t* was estimated as

5 $$\begin{aligned} v(t) = \frac{\sqrt{(x_{t} - x_{t-1})^{2} + (y_{t} - y_{t-1})^{2}}}{\Delta t}, \end{aligned}$$

where $(x_t, y_t)$ is the two-dimensional UWB-derived position and Δt=0.1 s is the sampling interval. Sustained inactivity (velocity <5 cm/second for ≥2 seconds) was classified as *resting*.

Spatial zones were annotated in arena coordinates using the lower-left corner of the arena as the origin. The food-access zone was defined as $0.10 \le x \le 0.20$ m and $0.10 \le y \le 0.20$ m, and occupancy within this zone for ≥1 s was classified as *feeding*. The bedding zone was defined as $0.80 \le x \le 1.00$ m and $0.40 \le y \le 0.60$ m. Occupancy within this bedding zone with velocity <5 cm/s for ≥1 s was classified as *burrowing*. Samples not meeting the criteria for *resting*, *feeding*, or *burrowing*, but showing locomotion between zones, were classified as *exploring*.

This discretization produced symbolic sequences required for probabilistic modeling, converting continuous UWB sensor data into categorical trajectories suitable for entropy, Bernoulli, and Markov analyses.

### Information-theoretic and probabilistic analysis

Deterministic metrics such as RMSE and SNR quantify positional fidelity but do not explain how degraded signals reshape uncertainty or alter sequential dynamics in behavior. To address this gap, we apply Shannon entropy to measure the dispersion of localization errors, Bernoulli modeling to estimate the probability of achieving behaviorally relevant accuracy thresholds, and Markov chains to capture transition structure between states. Together, these complementary analyses establish a physics-to-behavior framework in which impairments in UWB sensor signal quality manifest as systematic changes in behavioral variability.

#### Shannon entropy of localization errors

Uncertainty in error distributions was quantified as

6 $$\begin{aligned} H = -\sum _{i=1}^{n} p_i \log _2 p_i, \end{aligned}$$

where $p_i$ is the probability of an absolute localization error falling within the $i^{th}$ fixed-width error interval. In this study, localization errors were discretized into 20-mm intervals, defined as 0–20 mm, 20–40 mm, 40–60 mm, 60–80 mm, 80–100 mm, and 100–120 mm. The same binning scheme was applied to both LoS and NLoS conditions before computing Shannon entropy. Higher entropy corresponds to more dispersed and less predictable localization errors, while lower entropy indicates more concentrated and predictable localization.

To avoid redundancy with the Markov-chain entropy-rate formulation below, transition uncertainty was quantified using $H_{\text {rate}}$ (Eq. 12) rather than as a separate conditional-entropy statistic. This definition keeps the reported LoS–NLoS comparison tied to one Markov-chain metric, expressed in bits per transition.

#### Bernoulli modeling of localization success

Localization accuracy was reformulated as a binary success process:

7 $$\begin{aligned} X = {\left\{ \begin{array}{ll} 1, & \text {if } |e| \le \epsilon \\ 0, & \text {if } |e| > \epsilon , \end{array}\right. } \end{aligned}$$

where *e* denotes the absolute localization error relative to the ground truth, and ϵ=20 mm is the behavioral tolerance threshold.

The empirical success probability was then estimated as

8 $$\begin{aligned} \hat{p} = \frac{1}{N}\sum _{k=1}^{N} X_k, \end{aligned}$$

with *N* the total number of trials. A 95% confidence interval was computed using the normal approximation to the binomial distribution:

9 $$\begin{aligned} \hat{p} \pm 1.96 \sqrt{\frac{\hat{p}(1-\hat{p})}{N}}. \end{aligned}$$

This probabilistic formulation links technical accuracy (error magnitude) to statistical confidence in behavioral classification, enabling interpretable thresholds for LoS and NLoS conditions.

#### Markov chain modeling of trajectory dynamics

Sequential dependencies in movement were modeled using a first-order Markov chain:

10 $$\begin{aligned} P_{ij} = \Pr (X_{t+1} = j \,|\, X_t = i), \end{aligned}$$

where $X_t$ denotes the discrete behavioral state at time *t*, with states defined as resting, exploring, feeding, or burrowing, and $\textbf{P}$ is the row-stochastic transition matrix.

Long-term structure of trajectories was characterized by the stationary distribution:

11 $$\begin{aligned} \boldsymbol{\pi } \textbf{P} = \boldsymbol{\pi }, \quad \sum _i \pi _i = 1, \end{aligned}$$

where $\boldsymbol{\pi }$ represents the equilibrium probabilities of occupying each state.

Variability in movement dynamics was quantified by the entropy rate of the chain:

12 $$\begin{aligned} H_{\text {rate}} = -\sum _{i=1}^{n} \pi _i \sum _{j=1}^{n} P_{ij} \log _2 P_{ij}, \end{aligned}$$

where $\boldsymbol{\pi }$ denotes the stationary distribution of the transition matrix $\textbf{P}$, and $P_{ij}$ is the transition probability from state *i* to state *j*. This metric, which is the standard measure of the chain’s unpredictability, captures the average uncertainty of the next state. Higher $H_{\text {rate}}$ indicates more variable trajectories, while lower values reflect more stereotyped or constrained movement patterns.

### Markov chain model construction

States $s_i$ were defined as discrete behavioral categories, corresponding to resting, exploring, feeding, and burrowing. No separate spatial-zone Markov chain was modeled in the present analysis; spatial coordinates were used only to assign behavioral categories according to the operational criteria in Table 1. Transitions between behavioral states were estimated empirically as

13 $$\begin{aligned} P_{ij} = \frac{N(s_i \rightarrow s_j)}{\sum _{k=1}^{n} N(s_i \rightarrow s_k)}, \end{aligned}$$

where $N(s_i \rightarrow s_j)$ counts observed movements from state $s_i$ to $s_j$.

For an observed sequence $X_1,\dots ,X_T$, transition probabilities were estimated directly from empirical transition counts using Eq. 13. Long-term occupancy patterns were characterized by the stationary distribution, obtained as the dominant eigenvector of $\textbf{P}$. Temporal variability was quantified using the Markov-chain entropy rate and state-persistence probabilities, which were the reported dynamic metrics used to compare LoS and NLoS conditions.

**Table 1:** Operational definitions for behavioral states used in Markov modeling. States are defined using kinematic thresholds and spatial zones, enabling reproducible segmentation

State	Criteria
Resting	Velocity <5 cm/s maintained for ≥2 s.
Exploring	Locomotion between zones that did not satisfy the criteria for resting, feeding, or burrowing.
Feeding	Occupancy in the food-access zone ($0.10 \le x \le 0.20$ m, $0.10 \le y \le 0.20$ m) for ≥1 s.
Burrowing	Occupancy in the bedding zone ($0.80 \le x \le 1.00$ m, $0.40 \le y \le 0.60$ m) with velocity <5 cm/s for ≥1 s.

**Fig. 2:**
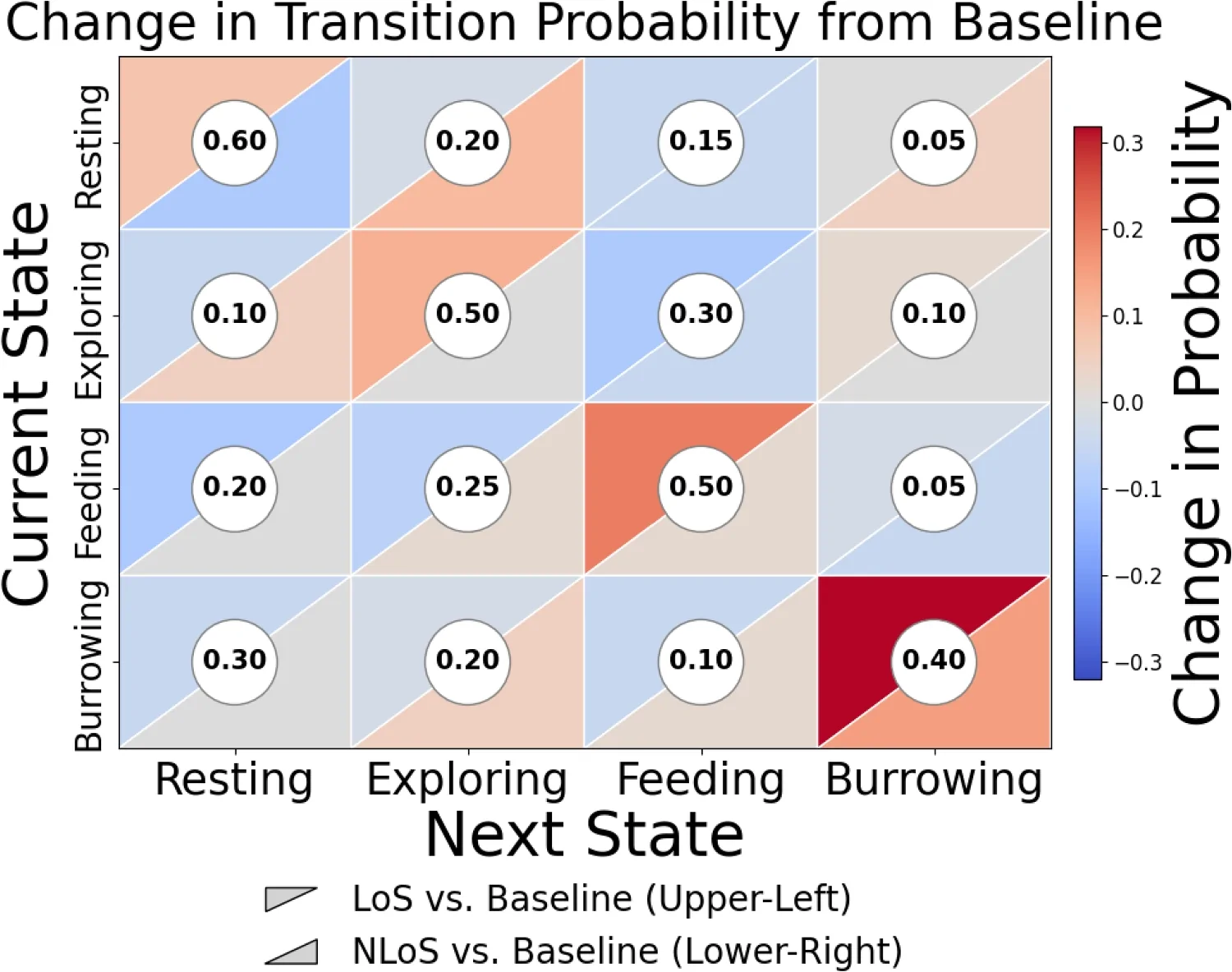
Split-cell difference heatmap illustrating the change in transition probabilities from the baseline. Each cell represents a single transition (e.g., resting to exploring). The *number* in the center of the cell is the absolute baseline probability. Each cell is split diagonally: the *color of the upper-left triangle* shows the change for the LoS condition, and the *color of the lower-right triangle* shows the change for the NLoS condition, according to the color bar on the right (*red* indicates an increase, *blue* indicates a decrease)

For the split-cell difference heatmap in Fig. 2, the baseline transition matrix was computed by pooling transition counts across all four sessions, including both LoS and NLoS recordings, to represent the average behavioral-transition structure of the reanalyzed dataset.

#### Prediction accuracy

Short-term predictive reliability was assessed via leave-one-session-out cross-validation. Accuracy was computed as

14 $$\begin{aligned} \textrm{Accuracy} = \frac{\mathrm {Correct\ Predictions}}{\mathrm {Total\ Transitions}} \times 100 \end{aligned}$$

Binomial confidence intervals were used to quantify uncertainty around prediction accuracy. Prediction accuracy was retained as the primary short-term model-performance metric because it directly quantifies the proportion of correctly predicted state transitions across held-out sessions.

#### Stationary distribution consistency

Long-term agreement between model-predicted and empirically observed occupancies was evaluated using Kullback–Leibler divergence:

15 $$\begin{aligned} D_{\textrm{KL}}(P \parallel Q) = \sum _i P(i) \log \frac{P(i)}{Q(i)} \end{aligned}$$

where *P* is the empirically observed stationary distribution and *Q* is the model-predicted stationary distribution. Kullback–Leibler divergence was retained as the primary distributional-agreement metric because it directly quantifies the discrepancy between empirical and model-predicted stationary occupancies.

#### Variability across sessions and recordings

To assess reproducibility, transition matrices were compared using normalized Frobenius distance:

16 $$\begin{aligned} d_F(\textbf{P}_1, \textbf{P}_2) = \frac{\Vert \textbf{P}_1 - \textbf{P}_2\Vert _F}{\Vert \textbf{P}_1\Vert _F} \end{aligned}$$

where $\textbf{P}_1$ and $\textbf{P}_2$ are two transition matrices being compared and $\Vert \cdot \Vert _F$ is the Frobenius norm.

Together, these analyses provide a multidimensional evaluation of model fidelity. Prediction accuracy assesses short-term model performance, Kullback–Leibler divergence tests long-term agreement between empirical and model-predicted stationary distributions, entropy rate reflects behavioral flexibility, and Frobenius-distance comparisons capture reproducibility across sessions and recordings. Integrating these complementary perspectives demonstrates the robustness of UWB-based probabilistic behavioral modeling under both LoS and NLoS conditions.

### Reproducibility and data-processing pipeline

To enhance reproducibility, the complete analysis workflow was organized as a stepwise pipeline. First, time-stamped two-dimensional position files (*t*, *x*, *y*) were imported and separated by signal condition (LoS or NLoS). Second, localization errors were computed relative to the synchronized video-derived ground truth and summarized using RMSE, empirical cumulative distribution functions, Bernoulli success probabilities, and Shannon entropy. Third, continuous trajectories were converted into symbolic behavioral-state sequences using the operational definitions in Table 1. Fourth, empirical transition matrices were estimated from consecutive state pairs and used to compute stationary distributions, entropy rate, persistence probabilities, prediction accuracy, Kullback–Leibler divergence, and Frobenius-distance comparisons. Finally, all summary tables and figures were generated from the processed files using the analysis scripts deposited in the OSF repository. This pipeline provides a direct path from tracking coordinates to the reported probabilistic behavioral metrics.

**Fig. 3:**
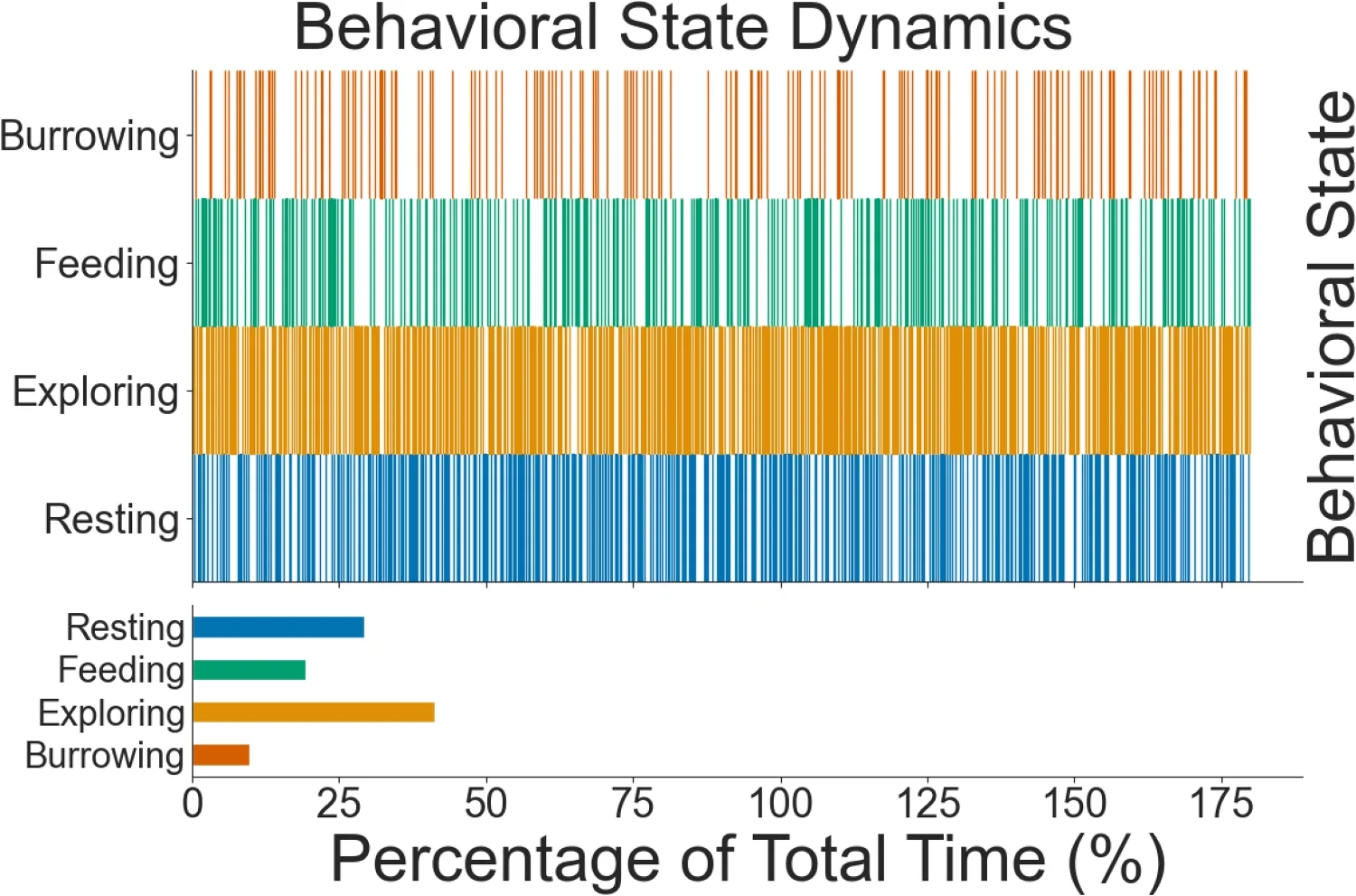
Comprehensive visualization of a behavioral session. *Top:* A timeline showing the sequence and duration of discrete behavioral states derived from UWB sensor tracking. *Bottom:* A summary bar chart quantifying the total percentage of time spent in each state, which represents the empirically observed stationary distribution

## Results

The Results are organized to follow the analytical workflow described in the Methods: behavioral-state transition structure is presented first, followed by graph-based transition visualization, information-theoretic and Bernoulli reliability analyses, UWB system-performance summaries, and the link between channel capacity and positioning accuracy.

### Behavioral transition matrices

First-order Markov chain models (Durbin et al., 1998) revealed structured organization in rodent activity. To visualize how LoS and NLoS conditions altered these patterns, we plotted the change in transition probabilities relative to the baseline in Fig. 2. This split-cell difference heatmap highlights the specific transitions that were most affected by signal quality. For context, the strongest transitions in the baseline data were *exploring* → *feeding* (P=0.30) and *feeding* → *resting* (P=0.20), forming a recurrent exploratory–feeding–resting cycle. Direct *resting* → *burrowing* transitions were rare (P<0.05), suggesting burrowing typically followed exploratory activity.

Figure 2 summarizes the baseline transition probabilities and the LoS–NLoS deviations from this baseline. Session-level consistency was high, with inter-session Frobenius norm differences <0.12, indicating reproducible transition patterns across recordings. Session-level variability was most pronounced in transitions involving *exploring* and *burrowing*, reflecting differences in exploratory drive and burrowing tendencies across recordings.

Stationary distributions derived from transition matrices closely matched empirically observed occupancies (mean Kullback–Leibler divergence <0.04), confirming that the models captured long-term behavioral tendencies. Because the present reanalysis was based on a single-rodent proof-of-concept dataset, reproducibility was evaluated descriptively using normalized Frobenius-distance comparisons across sessions. Inter-session distances were <0.12, providing a direct matrix-level measure of reproducibility, whereas the largest session-level variability was observed in transitions involving *exploring* and *burrowing*.

Entropy-rate values further distinguished LoS and NLoS conditions: under NLoS, the Markov-chain entropy rate increased to 1.28±0.04 bits per transition versus 1.10±0.03 bits per transition under LoS, reflecting greater next-state unpredictability when localization reliability decreased. Because entropy rate was summarized at the session level, this LoS–NLoS entropy-rate comparison was interpreted descriptively rather than as a primary inferential test.

Figure 3 provides a detailed visualization of the session, complementing the transition matrices. The top panel illustrates the short-term dynamics by showing the moment-to-moment behavioral sequences, while the bottom panel quantifies the long-term organization by summarizing the total time allocated to each state. Model-performance metrics across sessions are summarized in Table 2, which reports prediction accuracy, Kullback–Leibler divergence, and Markov-chain entropy-rate values used to evaluate reproducibility.

**Table 2:** Model performance across sessions. Prediction accuracy (PA), Kullback–Leibler divergence (KL), and Markov-chain entropy rate demonstrate stable reproducibility across sessions

Session	PA (%)	KL	Entropy rate (bits/transition)
1	83.1	0.035	1.21
2	81.7	0.041	1.18
3	82.9	0.038	1.23
4	84.0	0.033	1.20
Mean ± SD	82.9 ± 1.0	0.037 ± 0.003	1.21 ± 0.02

### State transition graphs

**Fig. 4:**
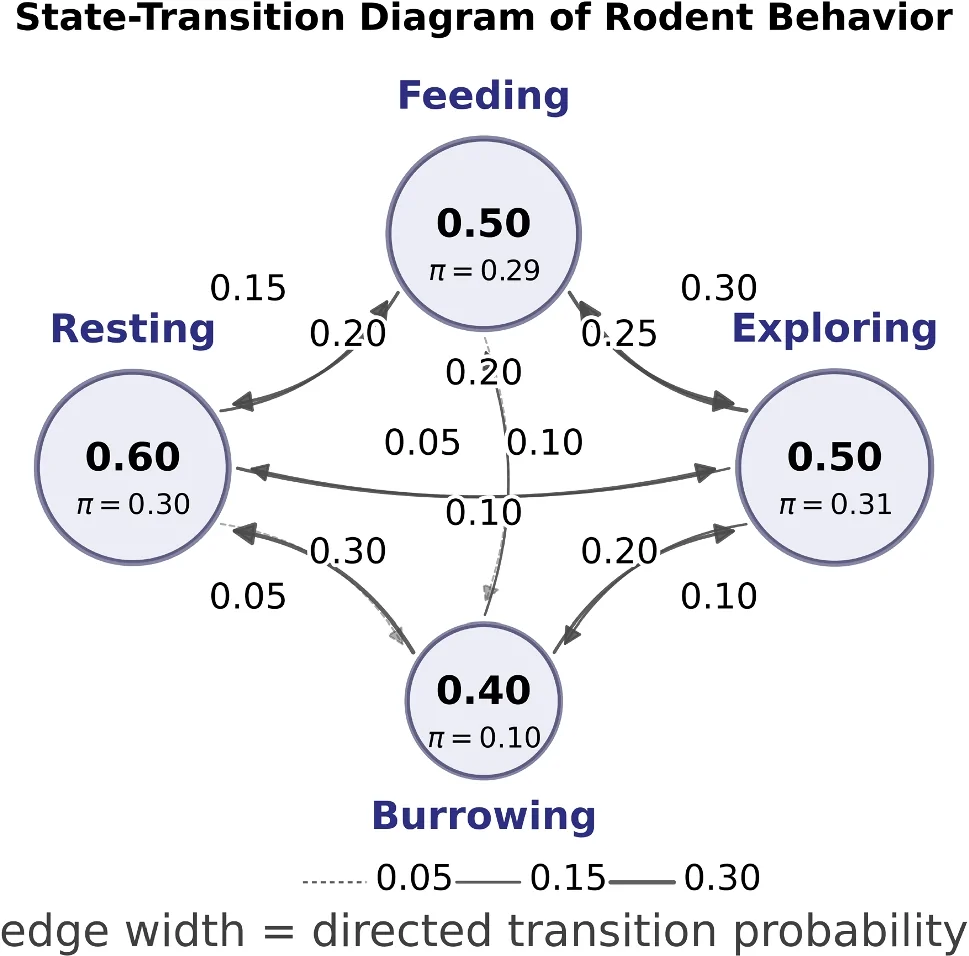
Revised state-transition diagram of rodent behavior. Directed *curved arrows* indicate unidirectional transition probabilities between behavioral states, with each probability label positioned next to its corresponding arrow to avoid ambiguity between reciprocal transitions. Node size is proportional to the stationary distribution, and the value inside each node represents the persistence probability for that state

Graph-based analyses provided a complementary view of behavioral connectivity, as shown in Fig. 4. Across sessions, *exploring* consistently emerged as a behavioral hub, with the highest centrality (mean 0.41), linking active and resource-directed states. Conversely, *burrowing* had the lowest connectivity (mean 0.12), consistent with its role as a more terminal state.

Graph density averaged 0.68±0.07, indicating a moderately interconnected behavioral network. Variability was greatest in *exploring* ↔ *burrowing* transitions, reflecting session-level differences in exploratory drive. Modularity analysis revealed stable clustering of *exploring* and *feeding* (Q=0.42±0.05), whereas *resting* and *burrowing* formed peripheral modules. The average shortest path length was 1.87±0.12, showing most states were accessible within two transitions.

### Information-theoretic and probabilistic results

**Fig. 5:**
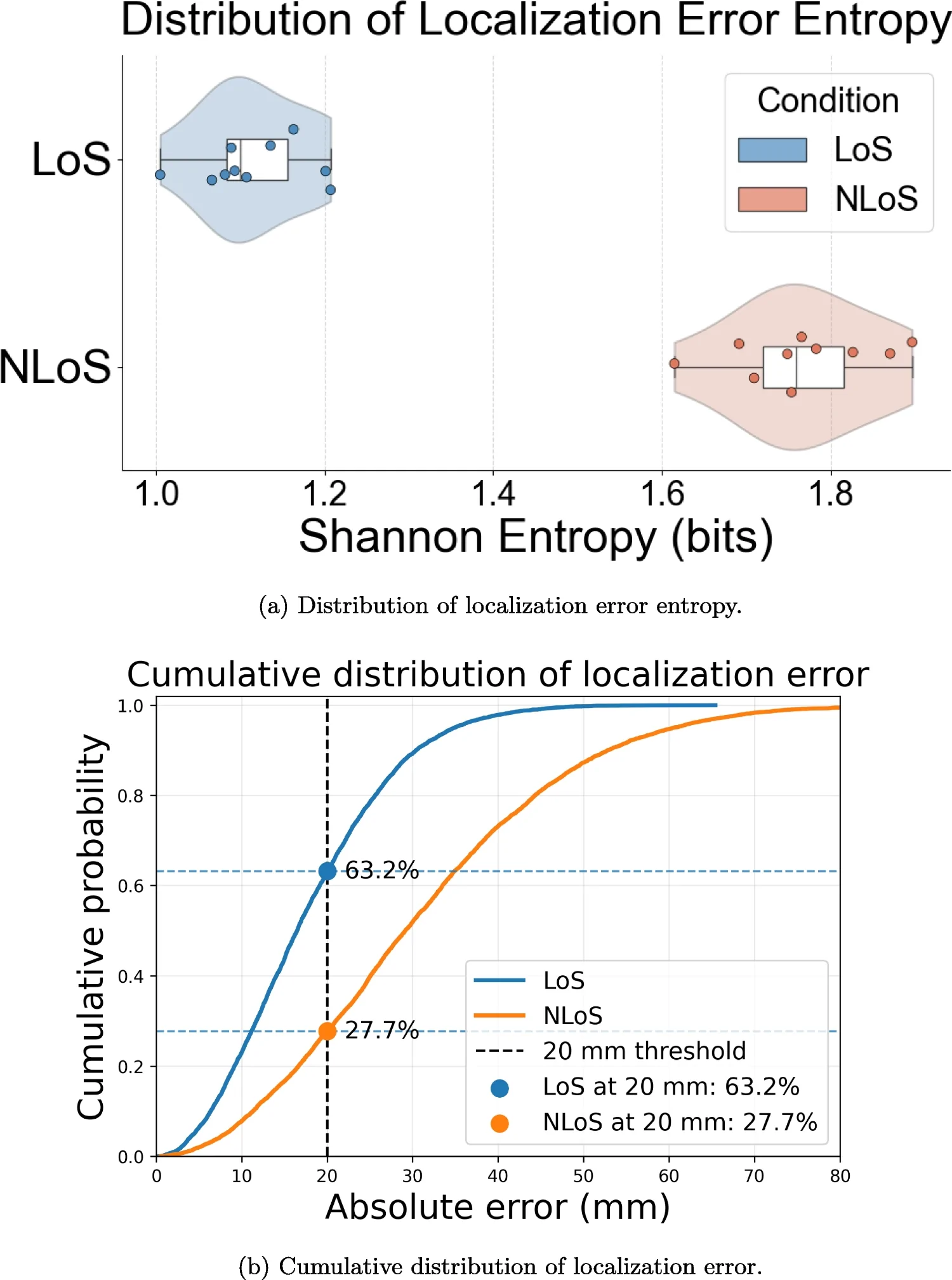
Comparison of information-theoretic measures under LoS and NLoS conditions. (**a**) The raincloud plot shows the full distribution of Shannon entropy. (**b**) The ECDF plot shows the cumulative probability of localization error, with the 20-mm threshold and empirical success probabilities explicitly annotated. At this threshold, the empirical success probability was 63.2% under LoS and 27.7% under NLoS, confirming reduced fine-scale localization reliability under NLoS

Information-theoretic analyses revealed systematic condition-dependent effects. The full distribution of localization error entropy under both conditions is visualized in Fig. 5a, showing a clear increase in both the mean and the variance of entropy from LoS (mean: 1.15 bits) to NLoS (mean: 1.78 bits). The full cumulative distribution of localization errors is shown in Fig. 5b. This ECDF plot, with the 20-mm threshold and annotated empirical success probabilities, visualizes the probability of achieving any given error threshold. For instance, at the 20-mm threshold indicated by the dashed line, the empirical success probability declined from 63.2% under LoS to 27.7% under NLoS. A two-proportion test confirmed that this reduction was statistically significant (p<0.001), with an absolute difference of 35.5 percentage points and a 95% confidence interval of 33.8–37.2 percentage points.

Markov analysis further indicated that state persistence decreased from 68% in LoS to 52% in NLoS (see Table 3), demonstrating more variable and fragmented sequences when tracking fidelity declined. A paired state-level comparison across the four behavioral categories showed that this persistence reduction was significant (t(3)=11.32, p=0.0015), with a mean decrease of 16.3 percentage points and a 95% confidence interval of 11.7–20.8 percentage points.

### UWB sensor system performance

To validate suitability for behavioral modeling, UWB sensor signal integrity and positioning accuracy were evaluated. Shannon–Hartley estimates (“Signal integrity modeling” section) indicated theoretical channel capacities exceeding 50 Mbps under both LoS and NLoS, despite reduced SNR in NLoS.

Empirical localization errors were approximately 20 mm under LoS and 35 mm under NLoS. Thus, LoS performance falls within the approximate range reported for high-end video tracking systems (15–25 mm), whereas NLoS performance shows reduced precision relative to that range while remaining suitable for coarse behavioral-state segmentation (Sayfoori et al., 2025).

**Table 3:** State persistence probabilities under LoS and NLoS conditions. Persistence (the diagonal of the transition matrix) decreased significantly under NLoS, indicating more fragmented behavior

State	Persistence (LoS)	Persistence (NLoS)
Resting	0.68	0.50
Exploring	0.62	0.50
Feeding	0.70	0.52
Burrowing	0.72	0.55
Average	0.68	0.52

### Theoretical vs. practical channel capacity and positioning

Although NLoS reduced SNR, practical link throughput remained sufficient for continuous high-frequency tracking. Errors under both conditions remained within ethologically relevant thresholds, enabling robust segmentation and probabilistic modeling. Table 4 summarizes the relationship between SNR, theoretical capacity, throughput, and observed error.

**Table 4:** Comparison of UWB sensor localization performance under LoS and NLoS conditions. SNR decreases under NLoS, reducing theoretical capacity and increasing error, while practical throughput (PL) remains stable

Condition	SNR (dB)	TC (Mbps)	PL (Mbps)	Error (mm)
LoS	20	3329.1	6.8	20
NLoS	10	1729.7	6.8	35

## Discussion

This study demonstrates that information-theoretic and probabilistic tools provide a deeper interpretation of UWB-based sensor tracking. The framework links physical signal quality to behavioral variability, showing how impairments in SNR cascade into uncertainty, reduced reliability, and altered state transitions. The results in Figs. 2–5 trace this physics-to-behavior pathway: from structured transition patterns and connectivity graphs to entropy- and Bernoulli-based quantification of uncertainty and reliability. The observed increase in Markov-chain entropy rate under NLoS conditions is likely driven by the corresponding decrease in state-persistence probabilities (the diagonal elements of the transition matrix) and the diffusion of probabilities to off-diagonal elements, reflecting more fragmented and unpredictable behavioral sequences.

Relative to our earlier Sensors Journal publication (Sayfoori et al., 2025), which established technical accuracy, the present work contributes a probabilistic analysis that transforms positional data into models of behavioral dynamics. Deterministic metrics provide point estimates of fidelity, whereas the probabilistic approach quantifies uncertainty, likelihood of success, and sequential dependencies, supporting reproducible behavioral phenotyping and aligning with calls for quantitative rigor in preclinical neuroscience (Luxem et al., 2022; Pereira et al., 2020).

Integrating Shannon–Hartley capacity analysis provides a mechanistic link: reductions in SNR limit channel capacity, degrade localization reliability, and elevate behavioral entropy (Molisch, 2011; Shannon, 1948). This connection unifies communication theory with ethology by showing how physical constraints manifest as higher-order variability in observed behavior.

Although the present study focuses on UWB-based localization, the proposed probabilistic modeling layer is not restricted to UWB signals. Once time-stamped two-dimensional coordinates are available, the same entropy, Bernoulli-threshold, stationary-distribution, and Markov-chain analyses can be applied to trajectories obtained from video-based tracking or other center-of-mass localization systems. In this sense, UWB provides the physical-layer case study used here to examine how signal degradation propagates into behavioral uncertainty, whereas the computational framework itself is sensor-agnostic and can support broader quantitative ethology workflows, including open-source video-tracking pipelines (Hsu & Yttri, 2021; Pereira et al., 2020).

This broader interpretation is consistent with prior spatial-behavior studies showing that center-of-mass trajectories can be transformed into quantitative descriptors of behavioral dynamics. In particular, León et al. demonstrated that spatial location, entropy-like measures, divergence, and multidimensional trajectory features can reveal structured behavioral organization in modified open-field settings and machine-learning-assisted behavioral analysis (León et al., 2020, 2021). These studies support the present work by situating our UWB-derived probabilistic metrics within a broader behavioral-analysis tradition rather than treating them only as engineering-performance outputs.

Beyond methodology, the framework addresses a practical need for quantitative, reproducible markers sensitive to dise-ase progression and pharmacological interventions. Elevated entropy in exploratory transitions may indicate fragmented search strategies in anxiety models, while changes in persistence probabilities could reveal drug-induced stabilization of feeding–resting cycles, highlighting translational potential. For instance, in a preclinical mouse model of a neurodegenerative disorder like Huntington’s disease, subtle behavioral fragmentation often precedes the onset of overt motor defi-cits. We hypothesize that our probabilistic metrics could serve as powerful early-stage biomarkers. An affected animal might exhibit a measurable increase in Markov-chain entropy rate as its movements become more disorganized, or a dec-rease in persistence probabilities for states such as *resting* or *feeding* as it struggles to maintain focused behaviors. Detecting such subtle changes long before they are visible through traditional scoring methods could provide a crucial window for evaluating the efficacy of therapeutic interventions.

### Limitations and future directions

#### Limitations

The first-order Markov assumption neglects higher-order dependencies and context, which can matter in memory- or decision-based tasks. Non-stationarity in social or perturbed environments may limit generalizability. Data sparsity for rare transitions (e.g., resting → burrowing) can destabilize estimates, motivating hierarchical Bayesian approaches or hidden Markov models (HMMs) (Sayfoori et al., 2025). The behavioral state segmentation relies on specific kinematic thresholds (e.g., speed and duration); the sensitivity of the probabilistic outputs to variations in these parameters was not explored and remains an area for future validation. Because this study reanalyzed a single-rodent localization dataset, session order and counterbalancing were not manipulated as independent experimental factors. Future prospective experiments should include multiple animals and counterbalance LoS and NLoS recording order across subjects to further separate signal-condition effects from possible order, fatigue, or habituation effects.

#### Future work

Extensions include multi-animal tracking with interaction modeling, and multimodal integration (e.g., EEG or cardiovascular signals) for joint neural–behavioral inference. Systematic benchmarking against computer vision pipelines (Hsu & Yttri, 2021; Pereira et al., 2020) will further clarify requirements for reproducibility, scalability, and annotation. Future studies could also test the framework’s performance in more complex, three-dimensional, or enriched environments, where behavioral states may be more diverse and challenging to classify.

## Conclusion

This study establishes a novel framework that transforms high-precision UWB sensor localization data into a rich source of probabilistic behavioral information. By integrating information-theoretic and Markovian analyses, we demonstrated that physical-layer signal impairments directly propagate to behavioral metrics. Specifically, under non-line-of-sight conditions, localization error entropy increased from 1.15 to 1.78 bits, the probability of achieving sub-20-mm accuracy declined from 63.2% to 27.7%, and behavioral state persistence decreased from 68% to 52%. The robustness of our framework was validated by the close match between model-predicted and empirical stationary distributions (KL divergence <0.04) and a predictive accuracy exceeding 82% across sessions.

By reframing UWB sensor trajectories as a probabilistic information source rather than a simple locator, our work provides an interpretable, reproducible, and scalable methodology for quantitative behavioral phenotyping. This enables the sensitive detection of phenotypic changes in disease models, the evaluation of pharmacological interventions, and longitudinal monitoring. Furthermore, the approach establishes a platform for multimodal neuroscience; integrating these probabilistic behavioral markers with physiological recordings, such as EEG, opens new avenues for mechanistic studies of brain–behavior relationships.

Taken together, these findings position this UWB-based sensor probabilistic approach as a precise, resource-efficient, and powerful complement to video-based systems, poised to accelerate translational research in preclinical and systems neuroscience.

## Open practices statement

The processed, de-identified data, materials, final figures, reproducibility tutorial, provenance manifest, and analysis scripts required to reproduce the reported figures and probabilistic metrics are available on the Open Science Framework at https://osf.io/s4ug9/. The study was not preregistered (Sayfoori and Cao 2025).

## Data Availability

Processed, de-identified datasets and all analysis code supporting this study are publicly available through the Open Science Framework (OSF): https://osf.io/s4ug9/. The repository includes final figures, a provenance manifest (docs/MANIFEST. json), a reproducibility tutorial (docs/TUTORIAL.md), processed data (errors, state sequences, transition matrices, and summary metrics), and analysis scripts required to regenerate the reported figures and probabilistic metrics (raw data not included) (Sayfoori and Cao 2025).
